# High male specific contribution of the X-chromosome to individual global recombination rate in dairy cattle

**DOI:** 10.1186/s12864-022-08328-8

**Published:** 2022-02-10

**Authors:** N. K. Kadri, J. Zhang, C. Oget-Ebrad, Y. Wang, C. Couldrey, R. Spelman, C. Charlier, M. Georges, T. Druet

**Affiliations:** 1 Unit of Animal Genomics, GIGA-R, 11 Avenue de l’Hôpital (B34), University of Liège, 4000 Liège, Belgium; 2 Animal Genomics, ETH Zürich, Universitätstrasse 2, 8092 Zürich, Switzerland; 3 Livestock Improvement Corporation Ltd, Private Bag 3016, 3240 Hamilton, New Zealand

## Abstract

**Background:**

Meiotic recombination plays an important role in reproduction and evolution. The individual global recombination rate (GRR), measured as the number of crossovers (CO) per gametes, is a complex trait that has been shown to be heritable. The sex chromosomes play an important role in reproduction and fertility related traits. Therefore, variants present on the X-chromosome might have a high contribution to the genetic variation of GRR that is related to meiosis and to reproduction.

**Results:**

We herein used genotyping data from 58,474 New Zealand dairy cattle to estimate the contribution of the X-chromosome to male and female GRR levels. Based on the pedigree-based relationships, we first estimated that the X-chromosome accounted for 30% of the total additive genetic variance for male GRR. This percentage was equal to 19.9% when the estimation relied on a SNP-BLUP approach assuming each SNP has a small contribution. We then carried out a haplotype-based association study to map X-linked QTL, and subsequently fine-mapped the identified QTL with imputed sequence variants. With this approach we identified three QTL with large effect accounting for 7.7% of the additive genetic variance of male GRR. The associated effects were equal to + 0.79, − 1.16 and + 1.18 CO for the alternate alleles. In females, the estimated contribution of the X-chromosome to GRR was null and no significant association with X-linked loci was found. Interestingly, two of the male GRR QTL were associated with candidate genes preferentially expressed in testis, in agreement with a male-specific effect. Finally, the most significant QTL was associated with *PPP4R3C*, further supporting the important role of protein phosphatase in double-strand break repair by homologous recombination.

**Conclusions:**

Our study illustrates the important role the X-chromosome can have on traits such as individual recombination rate, associated with testis in males. We also show that contribution of the X-chromosome to such a trait might be sex dependent.

**Supplementary Information:**

The online version contains supplementary material available at 10.1186/s12864-022-08328-8.

## Introduction

Recombination is a fundamental biological and evolutionary process. It creates genetic diversity among gametes and offspring by shuffling paternally and maternally inherited alleles, bringing favorable alleles together and separating them from deleterious alleles [1]. Recombination is also essential for reproduction as it ensures proper chromosome segregation during meiosis (e.g., [2]). An optimal number of crossovers (CO) per gamete is required for meiosis, and excessively low or high number of CO per gamete can result in fertility problems (e.g., [2]). Variation is nevertheless observed among species, sexes and individuals [1, 2]. For instance, a higher recombination rate (RR) has been measured in the homogametic sex in many species [3–7], although the opposite has also been observed in cattle [8, 9] and sheep [10]. Sex-specific distribution of CO have also been reported, with males presenting higher relative RR near telomeres [3, 8, 9, 11, 12].

Genetic studies have demonstrated that individual variation in global recombination rate (GRR), measured as the number of crossovers (CO) per gametes, is heritable in human [13], cattle [8, 9, 14], sheep [10, 15] and Drosophila [16]. Variants affecting GRR have been identified in several species including cattle [4, 8–10, 13–15, 17–19]. Several of these variants explained a large fraction of the genetic variance, up to 40%, suggesting that GRR has a rather oligogenic architecture [8, 10, 15, 18, 19].

The sex chromosomes play an important role in reproduction and fertility related traits (e.g., [20]). For instance, the human X-chromosome acquired and amplified testis-expressed gene families [21, 22], and both the human X and Y chromosomes gained a specialization for male reproduction [23]. Several examples of X-linked genes causing infertility or hybrid sterility, and also controlling meiosis, have been reported (e.g., [24, 25]). Further, X-linked loci could also explain the observed differences in GRR between sexes as suggested by Dumont and Payseur [19] and Dumont [26]. For these different reasons, the X-chromosome might make an important contribution to genetic variance of GRR. In mice, several X-linked loci associated with recombination have been identified (e.g., [19, 25]). However, only a few X-linked variants affecting GRR have been identified in other species, including human [see 26]. In cattle, GWAS have so far suggested a small contribution of the X-chromosome to the genetic variance of GRR [8, 9, 14]. Nonetheless, due to its specific inheritance pattern, the X-chromosome requires specific statistical tools and is sometimes ignored in GWAS (e.g., [27]).

We herein take advantage of the new bovine genome build [28], providing a significant improvement for the X-chromosome compared to the previous build, and an improved genetic map [29] to evaluate the contribution of the X-chromosome to male and female GRR in a dairy cattle population from New Zealand.

## Results

### Pedigree-based estimates of genetic variance components

First, using the pedigree-based relationship matrix, we partitioned the total phenotypic variance from male GRR into genetic, permanent environment and random residual variance without accounting for the contribution from the X-chromosome. The corresponding heritability and repeatability estimates were respectively 12.8% (± 1.3) and 16.5% (± 0.8). Next, we re-partitioned the phenotypic variance accounting for the contribution from the X-chromosome (X-specific part) in addition to the contribution from the autosomes by fitting these contributions as two distinct random effects. The heritability and repeatability estimates now increased to 15.8 (± 1.3) and 16.8 (± 0.8), respectively. The contribution of the X-chromosome was significant (*p* = 1.25e-7) and accounted for 4.7% (± 1.2) of the phenotypic variance and 29.9% (± 7.1) of the genetic variance. With the latter model, the contribution of the autosomes was reduced from 12.8 to 11.1%. The permanent environmental contribution was more impacted, decreasing from 3.8 to 1.0% suggesting that this effect could capture the X-chromosome effect when not included in the model. Subsequent analyses estimated that the genetic effects associated to the Y-chromosome captured respectively 0.5% (± 0.4) and 3.7% (± 3.1%) of the phenotypic and additive genetic variances, although its addition to the first model was non-significant (*p* = 0.054). The estimated contribution of the Y-chromosome was even lower, only 0.3% (± 0.3) of the total genetic variance, when the model also contained the genetic effect of the X-chromosome. Inclusion of the Y-chromosome effect was also less significant in that case (*p* = 0.261).

In females, the heritability and repeatability estimated with the first model, without sex-chromosome effects, were lower than in males, 5.8 (± 1.9) and 6.3 (± 2.0), respectively. The permanent environment variance also had a lower contribution to repeatability than in males. The inclusion of the X-chromosome additive genetic effects in females was non-significant and the variance associated with the X-chromosome was null.

### Variance associated with X-chromosome SNP effects

An alternative approach to estimate the contribution of the X-chromosome to the variance of individual GRR is to use a genomic relationship matrix (GRM). Here we used an equivalent SNP-BLUP approach. The genotype dosages, stored in the **X** matrix (see methods), were rescaled as for the computation of a GRM. The mean of diagonal elements from **XX**’ was 1.02 (SD 0.10) and 1.00 (SD 0.14) in males and females, respectively. Hence, the associated X-chromosome SNP-effect variance ${\sigma}_m^2$ corresponded to the variance of individual genetic effects of the X-chromosome. In males, this variance corresponded to 19.9 and 2.9% of the additive genetic variance and phenotypic variance, respectively. The resulting contribution was lower than the estimation from the pedigree-based relationships. The heritability was also slightly lower, 14.6%, whereas the repeatability, 16.8%, was equivalent to the value estimated with the pedigree-based model. These parameters were estimated with a REML algorithm as the AI-REML had convergence problems. We also used a Gibbs sampling approach to obtain confidence intervals, and estimates were consistent with to those from the REML analysis (Table S1 in Additional file 1). In females, the variance associated with the X-chromosome marker effects was null. Thus, these results confirm previous findings obtained with pedigree information, suggesting that the X-chromosome has a high contribution to genetic variance in males but a small contribution in females. Note that effects from rare alleles (MAF < 0.01%) were not captured by this approach.

### Haplotype- and sequenced-based association study

**Fig. 1:**
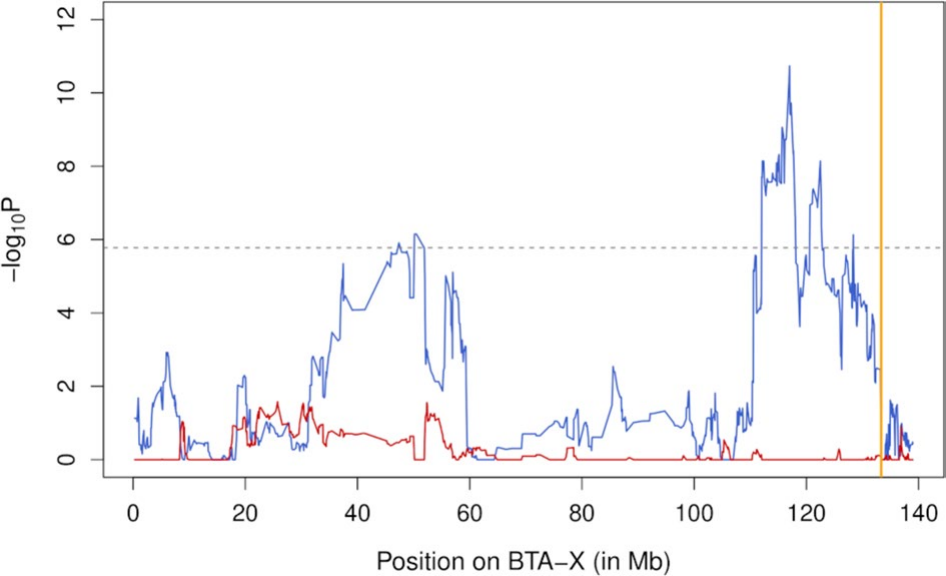
Haplotype-based association study for global recombination rate on the X-chromosome. The association study was performed in males (blue) and females (red). The gray line represents the genome-wide significance threshold and the position of the pseudo-autosomal boundary is represented in orange

**Fig. 2:**
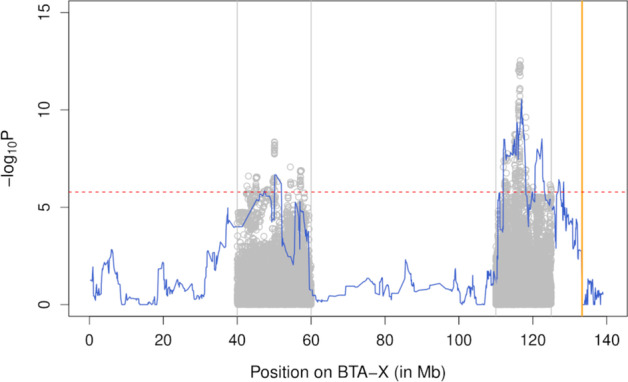
Association study for male global recombination rate on the X-chromosome. The association models include a haplotype-based association analysis (blue) and a sequenced-based single-point association in two regions harboring a QTL (gray). The red line represents the genome-wide significance threshold and the position of the pseudo-autosomal boundary is represented in orange

**Fig. 3:**
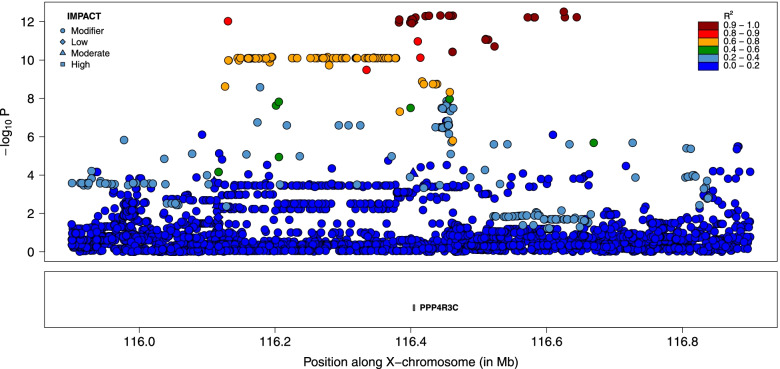
Fine-mapping of the distal QTL for GRR by sequence-based association analysis. Variants are colored according to their LD with the lead variant. The symbols are function of the predicted functional impact. The variants in dark red define the “LD-based set of candidate variants” assumed to encompass the causative variant

**Fig. 4:**
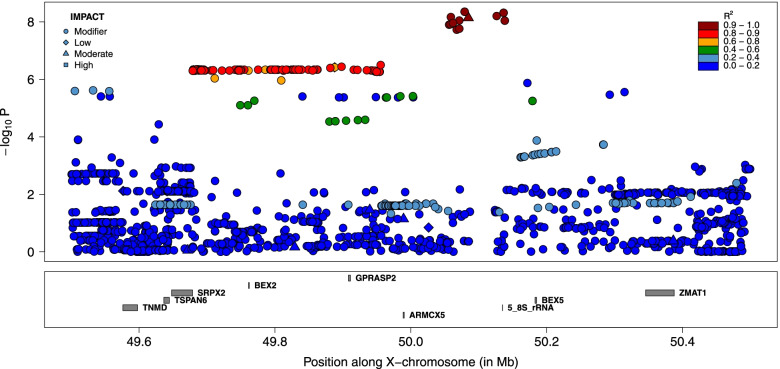
Fine-mapping of the proximal QTL for GRR by sequence-based association analysis. Variants are colored according to their LD with the lead variant. The symbols are function of the predicted functional impact. The variants in dark red define the “LD-based set of candidate variants” assumed to encompass the causative variant

Next, we carried out a haplotype-based association study to identify X-linked loci associated with variation in GRR. The results were in agreement with our first findings. Indeed, two QTL exceeding the significance threshold were identified in males (from positions 50.15 to 50.51 Mb, *P* = 7.1e-7; and at position 117.00 Mb, *P* = 1.8e-11) whereas no significant association was found in females (Fig. 1). To fine-map the two identified QTL, we performed a SNP-based association study with respectively 32,238 and 33,030 imputed sequence variants (see Material and Methods). The fine-mapping analyses were realized in windows of 20 Mb and 15 Mb centered around the most significant positions of the two QTL (respectively from positions 40 to 60 Mb, and from positions 110 to 125 Mb). In each window, the lead/top associated variant had higher significance than the haplotype-based analysis (Fig. 2). We defined LD-based sets of candidate variants for each QTL with SNPs and indels in high LD (r^2^ > 0.90) with the lead variant. These sets contained 35 (distal peak at chrX:116625344) and 13 variants (central peak at chrX:50079257), spanning approximately 276 and 82 kb (Figs. 3 and 4; Additional file 2).

**Fig. 5:**
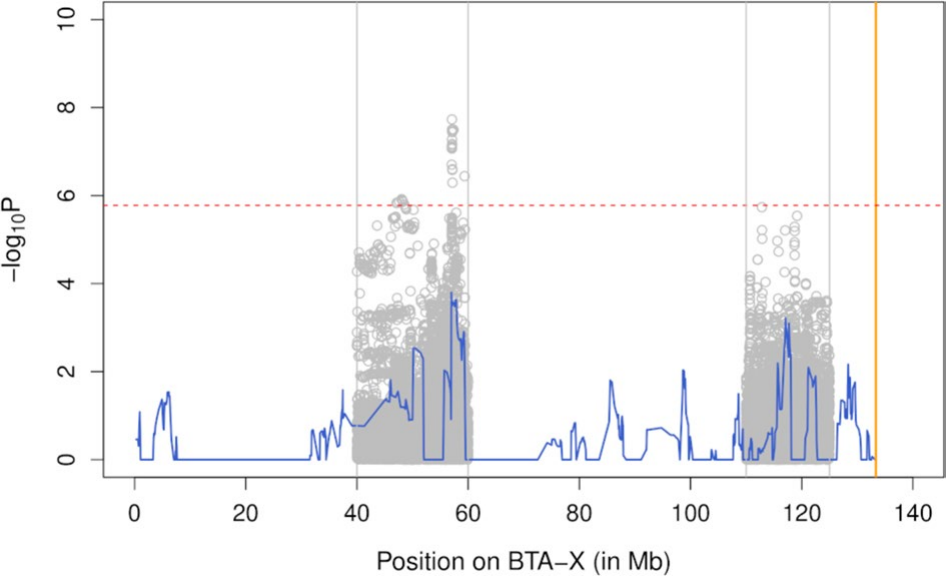
Conditional association study for male global recombination rate on the X-chromosome. The association models include a haplotype-based association analysis (blue) and a sequenced-based single-point association in two regions harboring a QTL (gray). In both association analyses, the two lead SNPs identified in the first sequenced-based association were included as covariates. The red line represents the genome-wide significance threshold and the position of the pseudo-autosomal boundary is represented in orange

**Fig. 6:**
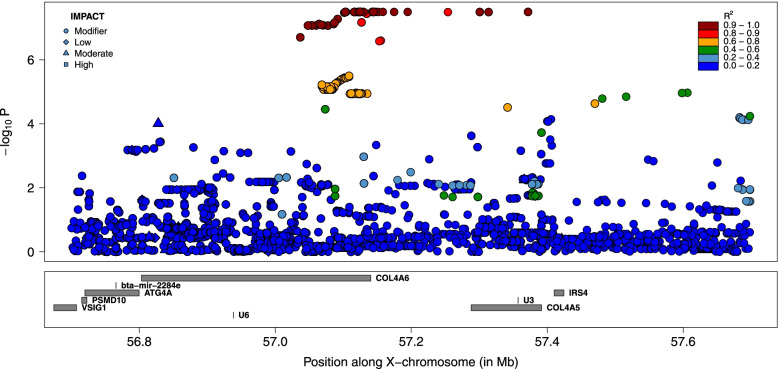
Peak of the sequenced-based association analysis conditional on the two primary lead SNPs. Variants are colored according to their LD with the lead variant. The symbols are function of the predicted functional impact. The variants in dark red define the “LD-based set of candidate variants” assumed to encompass the causative variant

We repeated both the haplotype-based and sequenced-based association analyses with the two lead-SNPs included in the model as fixed effects (Fig. 5). Although the haplotype-based analysis was no longer genome-wide significant in either interval, a secondary significant signal was obtained with the sequence-based analysis in the central peak (position 57.08 Mb, *P* = 7.1e-8), but not in the distal interval. The set of variants in high LD with the lead variant encompassed 37 variants in a 335 kb region (Fig. 6; Additional file 2). The lead variant was independent from the first lead variant identified in the interval at position chrX:50079257 (r^2^ = 0.001). Both haplotype-based and sequenced-based association analyses including the three lead variants as covariates were no longer significant in the interval (min *P* = 3.8e-1 and *P* = 4.4e-5 for the haplotype-based and sequenced-based analysis, respectively).

Functional annotation was used to determine which genes were associated to candidate variants (e.g., as coding variants in this gene, in upstream regions of these genes, etc.). Variants present in the three sets of LD-based candidate variants were associated to respectively only one, one and two genes (Additional file 2). In the most significant peak, the unique gene associated with the candidate variants was the *Protein Phosphatase 4 Regulatory Subunit 3C* or *PPP4R3C* (*ENSBTAG00000008511*), and the predicted functional impacts of the different variants were low (the set of candidate variants contained also a synonymous mutation). There was also a single gene (*ENSBTAG00000027978*) associated with the second set of LD-based candidate variants, that included a missense mutation M86I in *ENSBTAG00000027978* predicted to have a moderate effect with VEP and classified as tolerated with the SIFT score. This second set of LD-based candidate variants was also associated to two ribosomal RNA genes (5_8S_rRNA). Finally, in the third peak, variants were associated with *collagen type IV alpha 6 chain* (*COL4A6, ENSBTAG00000013760*) and *collagen type IV alpha 5 chain* (*COL4A5, ENSBTAG00000014575*).

### Contribution of identified loci to genetic variance in GRR

The effects of the identified genetic variants were estimated by simultaneously fitting the three newly identified variants on the X-chromosome in the LMM used for the sequenced-based association study. For GRR in males, the 10 previously identified autosomal variants (see Material and Methods) were also significant in our data set, and their effect ranged from 0.39 to 1.88 additional or fewer CO (Table 1). They accounted for 1.0 to 10.3% of the additive genetic variance (including genetic variance associated to autosomes and the X-chromosome). In total, the 10 autosomal variants captured respectively 45.5 and 64.7% of the additive genetic and the additive autosomal variances. The effects of the three newly identified variants from the X-chromosome on the GRR were large (Table 1), equal to respectively 0.78, 1.16 and − 1.18 CO for the alternate alleles of three leads SNPs (chrX:116625344, chrX:50079257 and chrX:57080199). The most significant variant (chrX:116625344) had moderate frequency (24.1%) whereas the two other variants were less frequent (around 5%). The variances associated with these effects were estimated as *pqa*^2^, where *p* and *q* are the allele frequencies and *a* is the additive effect. These variances represented respectively 3.4, 2.2 and 2.1% of the additive genetic variance despite the contribution of a single copy compared to autosomes carrying two alleles. Together, these three variants captured respectively 7.7 and 25.8% of the additive genetic and the X-chromosome genetic variances respectively. When all 13 GRR associated genetic variants were fitted in the model, the estimated polygenic variances associated with autosomes and the X-chromosome were reduced respectively by 43 and 36% (by 40% for the total additive genetic variance), confirming that they account for a large fraction of the additive genetic variance. Finally, none of the three newly identified variants had a significant effect on female GRR (*p* > 0.05).

**Table 1:** Frequency and effects on GRR (estimated jointly) of the variants previously identified on autosomes and the newly identified variants on the X-chromosome, and the corresponding proportion of additive genetic variance (associated with both autosomes and the X-chromosome) they account for

Fitted variants	*P*-value	Freq.	Effect	%Var
*HFM1 S1189L*	5.14 × 10^−06^	12.2%	−0.50	1.6%
*RNF212 P259S*	1.67 × 10^−28^	23.0%	0.98	10.3%
*RNF212 A77T*	2.40 × 10^− 18^	3.6%	−1.88	7.4%
rs381356614 (*RNF212B*)	6.72 × 10^− 18^	9.9%	1.06	6.1%
rs207682689 (*RNF212B*)	1.57 × 10^−08^	48.6%	−0.49	3.6%
rs437013002 (*RNF212B*)	1.99 × 10^− 03^	12.0%	−0.39	1.0%
*MLH3 N408S*	2.86 × 10^−18^	34.6%	0.70	6.7%
rs135941180	2.63 × 10^−11^	62.7%	−0.54	4.1%
*MSH5 R631Q*	2.24 × 10^− 11^	4.9%	−1.12	3.6%
rs1106661033 (*PRDM9*)	1.19 × 10^−04^	94.2%	−0.54	1.0%
ChrX 50,079,257 C/A (*ENSBTAG00000027978*)	1.13 × 10^− 09^	5.7%	1.16	2.2%
ChrX 116625345 TTC/−	2.68 × 10^−13^	24.1%	0.78	3.4%
ChrX 57,080,199 −/ATAT (*COL4A6*)	1.72 × 10^−08^	5.3%	−1.18	2.1%

## Discussion

We herein applied different approaches to estimate the contribution of X-chromosome loci to additive genetic variation in male and female GRR in cattle. The first approach was based on the pedigree-based relationship, the second on a highly polygenic model where X-chromosome SNP-effects are fitted simultaneously with the same variance, and a third approach relied on an X-chromosome wide association study that can reveal large effects loci. With the three approaches, the contribution of the X-chromosome to male GRR additive genetic variance was found to be substantial. Although the marker-based estimation of the percentage of genetic variance accounted by the X-chromosome was large (19.9%), it was lower than the pedigree-based estimation (29.9%). This difference might be due to the differences between expected and realized relationships that are larger when these are estimated for a single chromosome, such as the X-chromosome, rather than with all the autosomes [30]. Even higher deviations between expected and realized relationships were observed when comparisons were done exclusively for males and the for X-chromosome [30]. Use of realized relationships are expected to provide better estimators. The difference of estimated contributions might also result from relatively large standard errors associated with the estimators, indicating that it remained difficult to disentangle the respective contributions from autosomes, the X-chromosome and the permanent environmental effects. Three genome-wide significant QTL were identified on the X-chromosome and were fine-mapped with a sequenced-based association study. The genetic variance captured by the three identified loci represented respectively 7.7% of the additive genetic variance and 25.8% of genetic variation associated with the X-chromosome. This indicates that more variants on the X-chromosome affect GRR, but with lower effects or segregating at lower frequencies. We also estimated the contribution of the Y-chromosome to GRR but did not find evidence of large effects associated with specific male lineages. Interestingly, the variance of the X-chromosome and the Y-chromosome genetic effects were initially captured by the variance of the permanent environment effect, confirming that this effect might catch other genetic effects, with different inheritance patterns than autosomal additive effects, when these are not fitted in the model. Consequently, high observed levels of permanent environment variances, in past or future studies, might warrant further investigations. For instance, additional random effects such as those associated with sex-chromosomes or with transgenerational effects might be tested.

Contrasting results were obtained in females with the same approach as the contribution of the X-chromosome to female GRR was almost null. This discrepancy could be due to technical aspects, such as lower power in females where we have fewer records despite a larger number of parents, or such as the pedigree-based relationships **A** and **S** being more similar in females than in males (e.g., higher correlations between elements of the relationship matrices) (e.g., [30]), making it more difficult to disentangle the two effects. Nevertheless, the power was large enough to identify the variants present on the autosomes and affecting both male and female GRR [8]. Analysis of other populations used in Kadri et al. [8] showed similar patterns, with low non-significant contribution in females, albeit non-null, and several X-linked QTL in males (data not shown). Conversely, Ma et al. [9] found two QTL, one specific to each sex, in a large Holstein cattle pedigree realized on the previous bovine genome assembly. However, their study had higher association mapping power in females than in males. These associations on the X-chromosome were less significant than associations on the autosomes, suggesting moderate contributions to total variance. These observations nevertheless suggest that the contribution of the X-chromosome to the genetic variance of male or female GRR varies across populations. Variable contribution is also observed in other species. Dumont [26] reported that associations of X-linked variants with GRR remained rare in many species, including human, but also that large effect X-linked loci had been identified in mice [19, 25].

Our study also provides novel association between X-linked genes and recombination. In our most significant peak (lead SNP at position 116,625,345), the only gene associated with variants in high LD with the lead SNP is *PPP4R3C*, a paralogue of *PPP4R3A* and *PPP4R3B* that regulate protein phosphatase *Protein Phosphatase 4 Catalytic Subunit (PPP4C*) that is in turn associated with the DNA Double-Strand Break (DSB) repair pathway. Protein phosphatase PP4 have indeed been implicated in homologous recombination repair of DSB [31–33]. By catalyzing *RPA2* dephosphorylation, *PPP4C* allows the efficient recruitment of *RAD51* to chromatin [31]. Interestingly, *PPP4R3C* is preferentially expressed in testis in cattle, human and mice [34–36], and has been classified as male reproductive tract-specific genes by Roberston et al. [37]. Deficiency of *PPP4C* results in oligoasthenoteratospermia and sperm tail bending in mice [38]. These elements make *PPP43C* an interesting candidate gene and the association would confirm the importance of phosphorylation of repair proteins in the DSB repair pathway [33, 39]. However, no obvious candidate coding variant was identified in the candidate set of variants. The gene associated with variants from the second peak (lead SNP at position 50,079,257), *ENSBTAG00000027978*, is an orthologue of Prame (*Mus musculus*) and Pramel (*Rattus norvegicus*) from the *PRAME* (*Preferentially expressed antigen in melanoma*) family. The identity is however only moderate; 68.4% with *NM_029459* and 71.6% with *NM_001109368*. In human and mouse, *PRAME* expression is restricted primarily to the testis (in spermatogonia) [35, 36]. In agreement, the bovine *ENSBTAG00000027978* was more recently found to be mainly expressed in testis [34, 36]. The PRAME family has also been expanded by duplication, and the family is amplified on the Y-chromosome [40]. These genes are thus involved in male specific reproduction traits and have essential function in spermatogenesis. Interestingly, a missense variant *M86I* was in high LD with the lead SNP (r^2^ = 0.99), making it an excellent candidate causative variant. Finally, the third peak (lead SNP at position 57,080,119) encompasses *collagen type IV alpha 6 chain* (*COL4A6, ENSBTAG00000013760*) and *collagen type IV alpha 5 chain* (*COL4A5, ENSBTAG00000014575*) that are major structural components of basement membranes and expressed in several tissues, without direct link with recombination or male reproduction. The observation that two of the QTL were associated with genes specifically expressed in testis is compatible with male specific effects, providing further evidence of male specific effects on GRR in our population. Note that we cannot exclude that the causative variants were outside the defined credible sets. To obtain a set with a higher level of confidence, we could for instance include SNPs with a lower LD with the lead variant (r^2^ > 0.80). The implications of such changes can be visualized in Figs. 3, 4 and 6, and the additional SNPs are reported in Additional file 2. For the distal QTL (chrX:116625344; Fig. 3) and the secondary QTL (chrX: 57080199; Fig. 6), the credible sets would contain only 4 and 7 additional variants, without pointing to new genes. For the last QTL (chrX:50079257), a large cluster of 137 variants, ranging from positions 49,679,026 to 49,955,674, would be added (Fig. 4). This group of variants present however lower significance levels than those from the original credible set. Most of the additional variants are intergenic or in downstream/upstream regions of genes and are associated with six genes (Additional file 2), most without gene name. Some of these are preferentially expressed in testis and none of them as obvious relationship with meiotic recombination. Interestingly, the region also contained the *Brain Expressed X-Linked 2* (*BEX2*) gene, that regulates the level of PP2A regulatory subunit B and PP2A phosphatase activity that are related to meiotic recombination (the first QTL was also associated to protein phosphatase). However, no variant from this enlarged credible set was in intronic or exonic regions from *BEX2* or in its up/downstream regions.

In our previous study on GRR in cattle [8], most of the autosomal loci associated with GRR had shared effects in males and females. Here, we found sex-specific effects for loci on the X-chromosome, as for the two X-linked variants reported in Ma et al. [9]. In addition, Dumont and Payseur [19] suggested that if selection favors distinct recombination rates in both sexes, genetic variants affecting GRR might preferentially aggregate on the X-chromosome. The X-chromosome might thus play a role to generate the different recombination rates in males and females that are observed in several species [3–7, 9, 10].

The role of the X-chromosome in male GRR is also expected to be important because the X-chromosome has acquired testis-expressed genes [21, 23]. In human, both the X and Y-chromosomes have gained a specialization for male reproduction [23]. The X-chromosome has portions specialized for sperm production [22]. As a result, the X-chromosome is expected to harbor genes associated with male reproduction and with recombination. As an example, Yang et al. [24] found that *TEX11* regulates genome-wide recombination in mouse and that mutations in *TEX11* can cause infertility in males. In our study, the X-chromosome has specific effects on male GRR, and two of the candidate genes were such genes preferentially expressed in testis. These results are thus in line with the expectation that the X-chromosome harbors genetic modifiers affecting reproduction or meiosis related traits, and in particular in males.

## Material and methods

### Data

For the present study, we considered genotyping data from 58,474 cattle from New Zealand previously used to study recombination on autosomes [8] and the X-chromosome [29]. The population consisted of Holstein-Friesian (24%), Jersey (19%) and crossbred individuals (57%). The GRR phenotypes were previously estimated with LINKPHASE3 [41] based on 30,127 SNPs by Kadri et al. [8]. For each genotyped parent-offspring pair, the CO count corresponds to one record from the parent observed in one of its gametes. Parents with multiple genotyped offspring thus have multiple records observed in different gametes. In addition, imputed genotypes were available for a set of 11 genetic variants associated with GRR identified by Kadri et al. [8]. For the X-chromosome, haplotypes were estimated for 817 SNPs by Zhang et al. [29] after estimation of new sex-specific genetic maps with LINKPHASE3; 744 of these SNPs mapped to the X-specific part and 73 to the pseudo-autosomal region (PAR). LINKPHASE3 [41] relies on familial and linkage information and leaves some markers unphased. For further analysis, we kept individuals with GRR phenotypes from Kadri et al. [8], phased genotypes from Zhang et al. [29] and imputed genotypes for sequence variants (see below), resulting in a mapping cohort of 1962 males with 47,689 GRR records, and 5458 females with 7188 records. The pedigree of the 7420 parents with records and their ancestors included 20,785 individuals.

### Pedigree-based estimation of variance components

We estimated the genetic parameters associated with GRR in each sex separately with the following univariate linear mixed model (LMM):

$$\mathbf{y}=1\upmu +\mathbf{Pc}+{\mathbf{Z}}_{\mathrm{u}}\mathbf{u}+{\mathbf{Z}}_{\mathrm{p}}\mathbf{p}+\mathbf{e}$$

Where **y** is the vector of CO counts (e.g., GRR), μ is the overall mean effect, **c** is a vector of effects from the first four principal components of genetic variation (estimated with 30,127 autosomal SNPs), **u** is the vector of random polygenic effects normally distributed, i.e. **u** ~ *N*(0,**A** ${\sigma}_g^2$), where ${\sigma}_g^2$ is the additive genetic variance and **A** is the additive relationship matrix estimated from pedigree, **p** is the vector of random permanent environment effects normally distributed, i.e. **p** ~ *N*(0,**I** ${\sigma}_p^2$), where ${\sigma}_p^2$ is the variance of permanent environment effects and **I** is an identity matrix, **e** is the vector of random residual error terms normally distributed, i.e. **e** ~ *N*(0,**I** ${\sigma}_e^2$), where ${\sigma}_e^2$ is the residual variance. **P**, **Z**_u_ and **Z**_p_ are incidence matrices relating respective effects to phenotypes. The first four principal components were fitted to capture the breeds effects as we did in the first GWAS for GRR [8], they accounted for 8.8% of the variation.

The genetic variance associated with the X-chromosome (the X-specific part) was estimated by extending the LMM as follows:

$$\mathbf{y}=1\upmu +\mathbf{Pc}+{\mathbf{Z}}_{\mathrm{u}}\mathbf{u}+{\mathbf{Z}}_{\mathrm{p}}\mathbf{p}+{\mathbf{Z}}_{\mathrm{s}}\mathbf{s}+\mathbf{e}$$

where **s** is the vector of random additive genetic effects associated with the X-chromosome and normally distributed, i.e. **s** ~ *N*(0,**S** ${\sigma}_s^2$), where ${\sigma}_s^2$ is the additive genetic variance associated with the X-chromosome and **S** is the additive relationship matrix for the X-chromosome estimated from pedigree as in Fernando and Grossman [42], and reconstructed with codes developed in Druet and Legarra [30]. We realized a likelihood ratio test (LRT) comparing the two models to determine whether ${\sigma}_s^2$ was significantly different from 0.

For males, we could also estimate the contribution of the Y-chromosome. To that end, we assumed that sires transmitted their Y-chromosome effect to their sons. We identified 310 unique Y–chromosome lineages in our pedigree, 41 directly associated with records. Two models including a Y-chromosome genetic effect were tested:

$$\mathbf{y}=1\upmu +\mathbf{Pc}+{\mathbf{Z}}_{\mathrm{u}}\mathbf{u}+{\mathbf{Z}}_{\mathrm{p}}\mathbf{p}+{\mathbf{Z}}_{\mathrm{i}}\mathbf{i}+\mathbf{e}$$

and

$$\mathbf{y}=1\upmu +\mathbf{Pc}+{\mathbf{Z}}_{\mathrm{u}}\mathbf{u}+{\mathbf{Z}}_{\mathrm{p}}\mathbf{p}+{\mathbf{Z}}_{\mathrm{s}}\mathbf{s}+{\mathbf{Z}}_{\mathrm{i}}\mathbf{i}+\mathbf{e}$$

Where **i** is the vector of random Y-chromosome effects, normally distributed, **i** ~ *N*(0,**I** ${\sigma}_i^2$), where ${\sigma}_i^2$ is the additive genetic variance associated with the Y-chromosome and **Z**_i_ is the associated incidence matrix. These models were compared to previous models with a LRT.

Variance components were estimated with AIREMLF90 [43] and standard deviations of functions of genetic parameters (e.g., heritability, repeatability) were obtained by repeated sampling of parameters estimates from their asymptotic multivariate normal distribution [44].

### Estimation of variance associated with X-chromosome SNP-effects

We subsequently estimated the variance associated with the X-chromosome by using the genomic relationship matrix. We previously showed that such a GRM for the X-chromosome has a low dimensionality and is not of full rank [30]. Therefore, we proposed to use an equivalent SNP-BLUP approach (e.g., [45]) where X-chromosome SNP-effects (SNPs on the X-specific part) are simultaneously estimated. To that end, we fitted SNP as random effects in the following LMM:

$$\mathbf{y}=1\upmu +\mathbf{Pc}+{\mathbf{Z}}_{\mathrm{u}}\mathbf{u}+{\mathbf{Z}}_{\mathrm{p}}\mathbf{p}+\mathbf{Xm}+\mathbf{e}$$

Where **m** is the vector of M random marker (SNP) effects, normally distributed **m** ~ *N*(0,**I** ${\sigma}_m^2$), where ${\sigma}_m^2$ is the scaled SNP-effect variance. **X** is a matrix with the centered and scaled dosage of each SNP (one SNP per column) associated with the corresponding record (one line per record). The dosage for SNP *i* is estimated as the number of A alleles (0 or 1 in males as they are haploid on the X-chromosome, and 0, 1 or 2 in females). The dosages are centered by subtracting the A allele frequency, *p*_i_, in males and twice this value in females (see for instance [30]). Finally, the centered dosages are scaled by ${\sum}_{i=1}^M{p}_i\left(1-{p}_i\right)$ in males and $2{\sum}_{i=1}^M{p}_i\left(1-{p}_i\right)$ in females, similarly to the procedure from VanRaden [46]. The total additive genetic effect associated with the X-chromosome for individual *j*, *s*_*j*_, can be estimated as ${\sum}_{i=1}^M{x}_{ij}{m}_i$ where *m*_*i*_ is the effect of maker *i* (see for instance [47]) and *x*_*ij*_ is the dosage at marker *i* for individual *j*. As a result of the centering and scaling procedure, the variance of scaled SNP-effects ${\sigma}_m^2$ is expected to be equal to the variance of individual effects ${\sigma}_s^2$ [47]. This model was fitted separately in each sex.

The allele frequencies were estimated by counting one allele in males and two in females. We selected 585 SNPs with a minor allele frequency ≥ 0.01. The 585 SNP effects were fitted simultaneously with AIREMLF90 [43] and with a common variance. Parameters were also estimated with REMLF90 and GIBBSF90 [43] when AIREMLF90 did not converge properly.

### Haplotype-based association study

We performed a haplotype-based association study for loci on the X-chromosome. Genotypes were first phased based on familial and linkage information using LINKPHASE3 [38]. Unphased SNPs were subsequently phased with Beagle 4.1 [48], by exploiting linkage disequilibrium (LD) information. The haplotypes were then clustered according to their similarity with the model from Scheet and Stephens [49] as implemented in the PHASEBOOK program [50]. With this approach, haplotypes that are locally similar are grouped, at each marker position, into K, set to 40, haplotype clusters corresponding to the hidden states of the model. These two steps were done separately for the X-specific part, where the males were considered homozygotes, and the PAR.

Association between the position-specific haplotype clusters and individual GRR was then tested in each sex with the following LMM:

$$\mathbf{y}=\mathbf{X}\upbeta +\mathbf{Pc}+{\mathbf{Z}}_{\mathrm{u}}\mathbf{u}+{\mathbf{Z}}_{\mathrm{s}}\mathbf{s}+{\mathbf{Z}}_{\mathrm{h}}\mathbf{h}+\mathbf{e}$$

where **β** is a vector of fixed effects including the overall mean and the effect autosomal variants previously identified for their association with GRR by Kadri et al. [8], **h** is a vector of random haplotype clusters effects, normally distributed with variance ${\sigma}_h^2$, **X** and **Z**_h_ are incidence matrices. In the X-specific part, males and females are associated to respectively one or two haplotype cluster(s). The model included principal components and polygenic effects to account for population structure and cryptic relatedness, and also a sex-chromosome individual genetic effect to account for expected shared polygenic effects on the X-chromosome. For each trait (male and female GRR), we included in the model only those variants that were significantly associated to that specific trait. Respectively 10 and 8 autosomal variants were consequently fitted in males and females, including the following seven variants in both sexes *HFM1* S1189L, *RNF212* P259S, rs381356614 (*RNF212B*), rs207682689 (*RNF212B*), rs437013002 (*RNF212B*), *MLH3* N408S and rs1106661033 (associated with *PRDM9*). In addition, *RNF212* A77T, rs135941180 and *MSH5* R631Q were specifically fitted in males and rs210318688 (*MSH4*) in females.

As in Kadri et al. [8], the presence of a QTL was tested by comparing the likelihood of a model with versus without the random haplotype cluster effect with a LRT (distributed as a χ^2^ distribution with 1 df). We set the genome-wide significance threshold at 1.67e-6, after Bonferroni correction for 30,000 tests that include associations previously tested on autosomes [8].

### Sequenced-based association study

We used genotypes imputed for all parents in regions surrounding identified QTL. Imputed genotypes were available from a previous study relying on a reference panel of 1298 sequenced individuals. This imputation procedure realized with Beagle 5.1 [51] is fully described in Wang et al. [52] and was validated by a 10-fold cross-validation experiment. The coordinates from the two selected target regions were from 40 to 60 Mb and from 110 to 125 Mb. Respectively, 52,934 and 73,988 variants were imputed in these two regions. We kept for further analyses only variants with an estimated imputation accuracy r^2^ ≥ 0.80 (see distribution in Additional file 3: Fig. S1) and a MAF ≥ 0.01, resulting in 32,238 and 33,030 variants. We used the same LMM as in the haplotype-based association analysis but the haplotype effect was replaced by a regression on SNP allelic dosage. The association of this fixed effect with GRR was tested with a Z-test (see for instance [8]). For each of the fine-mapped QTL, we considered all the variants in high LD (r^2^ > 0.90) with the lead variant as the strongest candidate variants (LD-based set of candidate variants). Annotation of the variants was done with Variant Effect Predictor (VEP) v95.0 [53]. Information on gene expression for candidate genes was obtained from the Cattle Gene Atlas (http://cattlegeneatlas.roslin.ed.ac.uk/) [34], the human protein atlas (https://www.proteinatlas.org/) [35] and the Expression Atlas (https://www.ebi.ac.uk/gxa/home) [36].

## Supplementary Information

**Additional file 1: Table S1.** Estimation of genetic parameters with a REML and Gibbs sampling approach.

**Additional file 2.** Credible sets of variants in high LD with the lead SNPs for the three fine-mapped regions. The table reports the variants in high LD with the lead SNP and their functional annotation.

**Additional file 3: Figure S1.** Distribution of imputation accuracy.

## Data Availability

The data that support the findings of this study are available from Livestock Improvement Company (New Zealand) but restrictions apply to the availability of these data, which were used under license for the current study, and so are not publicly available. Data are however available from the authors upon reasonable request and with permission of the owners.

## References

[1] Stapley J, Feulner PG, Johnston SE, Santure AW, Smadja CM (2017). Variation in recombination frequency and distribution across eukaryotes: patterns and processes. Philos Trans R Soc B Biol Sci.

[2] Coop G, Przeworski M (2007). An evolutionary view of human recombination. Nat Rev Genet.

[3] Broman KW, Murray JC, Sheffield VC, White RL, Weber JL (1998). Comprehensive human genetic maps: individual and sex-specific variation in recombination. Am J Hum Genet.

[4] Chowdhury R, Bois PR, Feingold E, Sherman SL, Cheung VG (2009). Genetic analysis of variation in human meiotic recombination. PLoS Genet.

[5] Petkov PM, Broman KW, Szatkiewicz JP, Paigen K (2007). Crossover interference underlies sex differences in recombination rates. Trends Genet.

[6] Neff MW, Broman KW, Mellersh CS, Ray K, Acland GM, Aguirre GD (1999). A second-generation genetic linkage map of the domestic dog, *Canis familiaris*. Genetics.

[7] Muñoz M, Alves E, Ramayo-Caldas Y, Casellas J, Rodríguez C, Folch JM (2012). Recombination rates across porcine autosomes inferred from high-density linkage maps. Anim Genet.

[8] Kadri NK, Harland C, Faux P, Cambisano N, Karim L, Coppieters W (2016). Coding and noncoding variants in HFM1, MLH3, MSH4, MSH5, RNF212, and RNF212B affect recombination rate in cattle. Genome Res.

[9] Ma L, O’Connell JR, VanRaden PM, Shen B, Padhi A, Sun C (2015). Cattle sex-specific recombination and genetic control from a large pedigree analysis. PLoS Genet.

[10] Johnston SE, Bérénos C, Slate J, Pemberton JM (2016). Conserved genetic architecture underlying individual recombination rate variation in a wild population of Soay sheep (Ovis aries). Genetics..

[11] Liu EY, Morgan AP, Chesler EJ, Wang W, Churchill GA, Pardo-Manuel de Villena F (2014). High-resolution sex-specific linkage maps of the mouse reveal polarized distribution of crossovers in male germline. Genetics..

[12] Venn O, Turner I, Mathieson I, de Groot N, Bontrop R, McVean G (2014). Strong male bias drives germline mutation in chimpanzees. Science..

[13] Fledel-Alon A, Leffler EM, Guan Y, Stephens M, Coop G, Przeworski M (2011). Variation in human recombination rates and its genetic determinants. PLoS One.

[14] Sandor C, Farnir F, Hansoul S, Coppieters W, Meuwissen T, Georges M (2006). Linkage disequilibrium on the bovine X chromosome: characterization and use in quantitative trait locus mapping. Genetics..

[15] Petit M, Astruc J-M, Sarry J, Drouilhet L, Fabre S, Moreno CR (2017). Variation in recombination rate and its genetic determinism in sheep populations. Genetics..

[16] Kidwell MG (1972). Genetic change of recombination value in Drosophila melanogaster. I. Artificial selection for high and low recombination and some properties of recombination-modifying genes. Genetics..

[17] Kong A, Thorleifsson G, Stefansson H, Masson G, Helgason A, Gudbjartsson DF (2008). Sequence variants in the RNF212 gene associate with genome-wide recombination rate. Science..

[18] Kong A, Thorleifsson G, Frigge ML, Masson G, Gudbjartsson DF, Villemoes R (2014). Common and low-frequency variants associated with genome-wide recombination rate. Nat Genet.

[19] Dumont BL, Payseur BA (2011). Genetic analysis of genome-scale recombination rate evolution in house mice. PLoS Genet.

[20] Pacheco HA, Rezende FM, Peñagaricano F (2020). Gene mapping and genomic prediction of bull fertility using sex chromosome markers. J Dairy Sci.

[21] Bellott DW, Skaletsky H, Pyntikova T, Mardis ER, Graves T, Kremitzki C (2010). Convergent evolution of chicken Z and human X chromosomes by expansion and gene acquisition. Nature..

[22] Mueller JL, Skaletsky H, Brown LG, Zaghlul S, Rock S, Graves T (2013). Independent specialization of the human and mouse X chromosomes for the male germ line. Nat Genet.

[23] Bellott DW, Hughes JF, Skaletsky H, Brown LG, Pyntikova T, Cho T-J (2014). Mammalian Y chromosomes retain widely expressed dosage-sensitive regulators. Nature..

[24] Yang F, Silber S, Leu NA, Oates RD, Marszalek JD, Skaletsky H (2015). TEX 11 is mutated in infertile men with azoospermia and regulates genome-wide recombination rates in mouse. EMBO Mol Med.

[25] Balcova M, Faltusova B, Gergelits V, Bhattacharyya T, Mihola O, Trachtulec Z (2016). Hybrid sterility locus on chromosome X controls meiotic recombination rate in mouse. PLoS Genet.

[26] Dumont BL (2017). X-chromosome control of genome-scale recombination rates in house mice. Genetics..

[27] Qanbari S, Wittenburg D (2020). Male recombination map of the autosomal genome in German Holstein. Genet Sel Evol.

[28] Rosen BD, Bickhart DM, Schnabel RD, Koren S, Elsik CG, Tseng E (2020). De novo assembly of the cattle reference genome with single-molecule sequencing. Gigascience..

[29] Zhang J, Kadri NK, Mullaart E, Spelman R, Fritz S, Boichard D (2020). Genetic architecture of individual variation in recombination rate on the X chromosome in cattle. Heredity..

[30] Druet T, Legarra A (2020). Theoretical and empirical comparisons of expected and realized relationships for the X-chromosome. Genet Sel Evol.

[31] Lee D-H, Pan Y, Kanner S, Sung P, Borowiec JA, Chowdhury D (2010). A PP4 phosphatase complex dephosphorylates RPA2 to facilitate DNA repair via homologous recombination. Nat Struct Mol Biol.

[32] Lee D-H, Goodarzi AA, Adelmant GO, Pan Y, Jeggo PA, Marto JA (2012). Phosphoproteomic analysis reveals that PP4 dephosphorylates KAP-1 impacting the DNA damage response. EMBO J.

[33] Liu J, Xu L, Zhong J, Liao J, Li J, Xu X (2012). Protein phosphatase PP4 is involved in NHEJ-mediated repair of DNA double-strand breaks. Cell Cycle.

[34] Fang L, Cai W, Liu S, Canela-Xandri O, Gao Y, Jiang J (2020). Comprehensive analyses of 723 transcriptomes enhance genetic and biological interpretations for complex traits in cattle. Genome Res.

[35] Uhlén M, Fagerberg L, Hallström BM, Lindskog C, Oksvold P, Mardinoglu A (2015). Tissue-based map of the human proteome. Science..

[36] Kapushesky M, Emam I, Holloway E, Kurnosov P, Zorin A, Malone J (2010). Gene expression atlas at the European bioinformatics institute. Nucleic Acids Res.

[37] Robertson MJ, Kent K, Tharp N, Nozawa K, Dean L, Mathew M (2020). Large-scale discovery of male reproductive tract-specific genes through analysis of RNA-seq datasets. BMC Biol.

[38] Han F, Dong MZ, Lei WL, Xu ZL, Gao F, Schatten H (2021). Oligoasthenoteratospermia and sperm tail bending in PPP4C-deficient mice. Mol Hum Reprod.

[39] Shrivastav M, De Haro LP, Nickoloff JA (2008). Regulation of DNA double-strand break repair pathway choice. Cell Res.

[40] Chang T-C, Yang Y, Yasue H, Bharti AK, Retzel EF, Liu W-S (2011). The expansion of the PRAME gene family in Eutheria. PLoS One.

[41] Druet T, Georges M (2015). LINKPHASE3: an improved pedigree-based phasing algorithm robust to genotyping and map errors. Bioinformatics..

[42] Fernando RL, Grossman M (1990). Genetic evaluation with autosomal and X-chromosomal inheritance. Theor Appl Genet.

[43] Misztal I, Tsuruta S, Strabel T, Auvray B, Druet T, Lee DH (2002). BLUPF90 and related programs (BGF90). Proceedings of the 7th world congress on genetics applied to livestock production.

[44] Meyer K, Houle D (2013). Sampling based approximation of confidence intervals for functions of genetic covariance matrices. Proc. Assoc. Advmt. Anim. Breed. Genet.

[45] Strandén I, Garrick DJ (2009). Derivation of equivalent computing algorithms for genomic predictions and reliabilities of animal merit. J Dairy Sci.

[46] VanRaden PM (2008). Efficient methods to compute genomic predictions. J Dairy Sci.

[47] Yang J, Benyamin B, McEvoy BP, Gordon S, Henders AK, Nyholt DR (2010). Common SNPs explain a large proportion of the heritability for human height. Nat Genet.

[48] Browning SR, Browning BL (2007). Rapid and accurate haplotype phasing and missing-data inference for whole-genome association studies by use of localized haplotype clustering. Am J Hum Genet.

[49] Scheet P, Stephens M (2006). A fast and flexible statistical model for large-scale population genotype data: applications to inferring missing genotypes and haplotypic phase. Am J Hum Genet.

[50] Druet T, Georges M (2010). A hidden Markov model combining linkage and linkage disequilibrium information for haplotype reconstruction and quantitative trait locus fine mapping. Genetics..

[51] Browning BL, Zhou Y, Browning SR (2018). A one-penny imputed genome from next-generation reference panels. Am J Hum Genet.

[52] Wang Y, Tiplady K, Johnson TJJ, Harland C, Keehan M, Lopdell T (2021). Investigating the accuracy of imputing variants on chromosome X in admixed dairy cattle using the ARS-UCD1.2 assembly of the bovine genome. Proceedings from the 38th International Society for Animal Genetics Virtual Conference.

[53] McLaren W, Gil L, Hunt SE, Riat HS, Ritchie GR, Thormann A (2016). The ensembl variant effect predictor. Genome Biol.

