# Supplementary material for: High male specific contribution of the X-chromosome to individual global recombination rate in dairy cattle
Source: BMC Genomics. 2022 Feb 10;23:114. doi: 10.1186/s12864-022-08328-8 (PMC8832838; doi:10.1186/s12864-022-08328-8)
Supplement: Supplementary file 1 — Additional file 1: Table S1. Estimation of genetic parameters with a REML and Gibbs sampling approach. [file 12864_2022_8328_MOESM1_ESM.docx]

Table S1. Estimation of genetic parameters in a model including X-chromosome SNP effects with a REML algorithm and a Gibbs sampling approach. Five different chains were run for 130,000 iterations with a burn-in of 30,000 samples. We saved parameters every 50 samples.

| Estimation method | Percentage variance explained by X-chromosome SNPs (in%) | | Genetic parameters | |
| --- | --- | --- | --- | --- |
|  | From total variance | From genetic variance | Heritability^1^ | Repeatability^1^ |
| REML | 2.9 | 19.9 | 14.6 | 16.8 |
| REML^2^ | 2.9 | 20.1 | 14.6 | 16.8 |
| Gibbs – run1 | 3.1 ± 0.1 | 20.6 ± 4.1 | 14.9 ± 1.3 | 17.0 ± 0.8 |
| Gibbs – run2 | 3.1 ± 0.1 | 20.9 ± 4.1 | 14.8 ± 1.2 | 17.0 ± 0.8 |
| Gibbs – run3 | 3.1 ± 0.1 | 20.9 ± 4.0 | 14.7 ± 1.2 | 17.0 ± 0.8 |
| Gibbs – run4 | 3.1 ± 0.1 | 21.0 ± 4.0 | 14.8 ± 1.2 | 17.0 ± 0.8 |
| Gibbs – run5 | 3.1 ± 0.1 | 20.8 ± 4.0 | 14.9 ± 1.2 | 17.0 ± 0.8 |

^1^Heritability and repeatability estimates included the additive genetic contribution of the autosomes and the X-chromosome SNPs; ^2^A second REML was run with different starting values.

Figure S1. Distribution of imputation accuracy.
