# Supplementary figures and images for: High male specific contribution of the X-chromosome to individual global recombination rate in dairy cattle
Source: BMC Genomics. 2022 Feb 10;23:114. doi: 10.1186/s12864-022-08328-8 (PMC8832838; doi:10.1186/s12864-022-08328-8)

Distribution of imputation accuracy

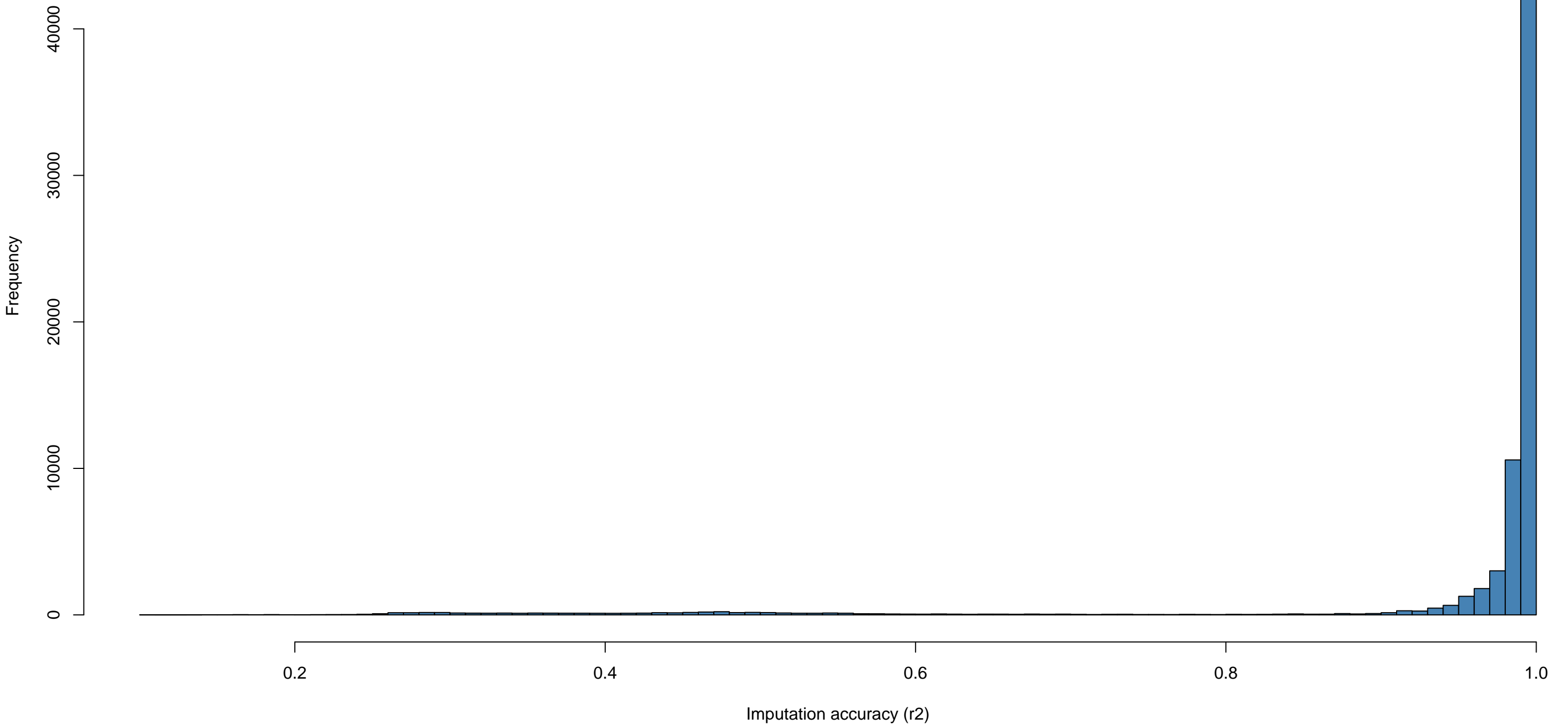

Supplement: Supplementary file 3 — Additional file 3: Figure S1. Distribution of imputation accuracy. [file 12864_2022_8328_MOESM3_ESM.pdf]
